# Combined Action of Antibiotics and Bacteriocins against Vancomycin-Resistant Enterococci

**DOI:** 10.3390/microorganisms10071423

**Published:** 2022-07-14

**Authors:** Jorge Enrique Vazquez Bucheli, Joanna Ivy Irorita Fugaban, Wilhelm Heinrich Holzapfel, Svetoslav Dimitrov Todorov

**Affiliations:** 1 Human Effective Microbes Laboratory, Department of Advanced Convergence, Handong Global University, Pohang 37554, Korea; jorge_jbv@hotmail.com (J.E.V.B.); wh@woodapple.net (W.H.H.); 2 ProBacLab, Department of Advanced Convergence, Handong Global University, Pohang 37554, Korea; jofu@food.dtu.dk

**Keywords:** bacteriocins, vancomycin-resistant enterococci (VRE), lactic acid bacteria (LAB)

## Abstract

Antibiotics have been one of the most important discoveries in the area of applied medical microbiology; however, as a result of various factors, we are currently facing a dramatic and relatively dangerous increase in the number of cases of antibiotic resistance, and the need for new types of antimicrobials continues to grow. New approaches are needed to combat antibiotic-resistant pathogens. Bacteriocins, as part of the group of antimicrobial peptides, can be considered as alternatives and/or complements to known antibiotics. Their narrow spectra of activity can be explored for the control of various pathogens, such as vancomycin-resistant enterococci (VRE), as single therapies or in combination with known antibiotics. In the present study, we isolated bacteriocins from different lactic acid bacteria (LAB) strains, including *Enterococcus* and *Pediococcus*, and explored the possible synergistic inhibition of growth by bacteriocins and vancomycin. It was observed in the growth dynamics with previously selected VRE strains that the bacteriocins had a high specificity and a promising inhibitory effect against the VRE strains, and these results were validated by a propidium iodide viability test using flow cytometry. The data obtained indicate that the selected bacteriocins can be used to control VRE in the food industry or even as an alternative treatment to combat infections with antibiotic-resistant bacteria.

## 1. Introduction

Antibiotic resistance is an emerging public health problem. This has triggered the realization of an urgent need for the development of new antimicrobials and, concomitantly, also the development of rapid and sensitive approaches for the identification of representative pathogens and mechanisms of antibiotic resistance [1]. Since the discovery of antibiotics and their commercialization, the scientific community also invariably evaluated the different negative aspects of the clinical application of antibiotics, resulting in the development of antibiotic-resistant strains and their predominant spread, especially in the hospital environment [2]. The presence of antibiotic-resistance genetic determinants is a natural phenomenon; however, and therefore, the irresponsible use of these agents from the second half of the 20th century accelerated the selective pressure of the antibiotic-resistant mutants and their survival and establishment as the predominant representatives in several ecosystems. In the recent past, antibiotics were applied at large scale, not just for the treatment of infectious diseases but also frequently and unnecessarily for the prevention of potential medical complications as growth promotors and for preventive treatment in the veterinary sector and in agricultural practices to increase production yields. The uncontrolled release of antibiotics in the environment contributed to the reduction of several human and animal pathogens; however, the lack of regulations on their use had negative consequences in the preselection and survival of antibiotic-resistant mutants and allowed them to predominate in the environment [3]. A similar scenario is typical of the current hospital environment, where multidrug-resistant bacterial pathogens have passively been preselected by the constant application of antibiotics, thus developing into a serious health threat. Among these resistant pathogens, vancomycin-resistant enterococci (VRE) and methicillin-resistant *Staphylococcus aureus* (MRSA), are recognized worldwide as bacteria of major health concern [4].

Bacteriocins, a non-posttranslational-modified antimicrobial peptide produced via ribosomal machinery of the bacterial cells [5] were suggested as a potential alternative and/or addendum to conventional antibiotics [6,7]. The narrow antimicrobial spectrum of bacteriocins is a characteristic that gives them an advantage in combating human and animal pathogens [8]. Considered safe, bacteriocins are characterized by a general low toxicity [7,9]. Their application was originally explored as bio-preservatives, and nisin, in particular, is approved in several countries for application in the food industry [10,11]. However, in the last decade, scientific interest has increasingly focused on the application of bacteriocins as an alternative and/or additive to antibiotics in the combat against emerging pathogens in human and veterinary practices. The applications of native and modified bacteriocins have been proposed as alternative treatments for *Mycobacterium tuberculosis* [12], MRSA [13], and VRE [14,15,16]. Moreover, the combined application of bacteriocins and antibiotics or essential oils were proposed as an alternative for the reduction of the required dose of antibiotics for application in human and veterinary medicine [12,17]. Will bacteriocins be the next generation antimicrobials in the treatment of relevant human and animal pathogens? However, related to the application of chemical preservatives in the food industry, a concern has been raised about the possible use of sublethal doses of non-antibiotic antimicrobials and their role in increasing MIC for antibiotics [17]. Questions on possible indirect interactions are of particular concern in view of potentially increasing problems with antibiotic resistance. Do bacteriocins need to be regarded as safe antimicrobials, indifferent to the antibiotic resistance phenomena, and could they even be considered as alternatives and maybe as new generation “antibiotics”? Already Heilbronner et al. [18] suggested that bacteriocins can be considered safe, and due to their effective antimicrobial activity, they could be attractive alternatives for precision therapy and prevention of bacterial infection. Moreover, the correct answer might be that we would need to predict the possible negative consequences, explore these possible challenging scenarios, and attempt to perform deeper research to avoid any future negative consequences. The aim of this study was to explore the possibility of effective combat of VRE by applying bacteriocins and to evaluate the safe applications of bacteriocins and their influence on the development of antibiotic resistance in VRE strains.

## 2. Materials and Methods

### 2.1. Differentiation and Identification of VRE Strains and Detection of the Type of Vancomycin Resistance

Vancomycin-resistant enterococci (VRE), previously isolated from hospital patients and fully characterized, were kindly provided by the Laboratory of Antibiotics (kindly provided from Prof. Kwak, Handong Global University, Pohang, Republic of Korea). In the present study, we have differentiated the VRE based on repPCR with primer (GTG)_5_, according to Valledor et al. [19]; these were also screened for the presence of vancomycin-resistant genes, including *van*A, *van*B, *van*C, *van*D, *van*E, and *van*G, according to Valledor et al. [19]. Further identification of these VRE strains was based on 16S rRNA partial gene sequencing at SolGent Analysis Service (Daejeon, Republic of Korea) as a commercial service, in addition to further biochemical and physiological tests, as recommended by Bergey’s Manual of Systematic Bacteriology [20].

The VRE strains were cultured in 10 mL MRS broth (Difco, Franklin Lakes, NJ, USA) at 37 °C for 24 h; the cells were harvested by centrifugation (4000× *g*, 10 min, 4 °C), and the DNA was extracted using ZR Fungal/Bacterial DNA Kit (Zymo Research, Irvine, CA, USA), according to the manufacturer’s recommendations. The purity and concentration of the DNA were evaluated spectrophotometrically on SPECTROstar Nano nanodrop (BMG LABTECH, Ortenberg, Germany). All PCR reactions were performed in a Veriti 96-well Thermal Cycler, Applied Biosystems (Thermo Scientific, Waltham, MA, USA). The generated amplicons were separated and visualized on 1.0–2.0% agarose (Sigma-Aldrich, St. Louis, MO, USA) in the presence of 0.02 μL mL^−1^ of SYBR^®^ Safe (Thermo Scientific) in 1× TAE buffer at 100 V for 1 h (GH-200 Genera Biosystems, Victoria, Australia; Elite 300 Plus Power Supply, Wealtec Bioscience Co., Ltd., Taipei, Taiwan). The gel was visualized using the Omega Lum™G gel documenter (Aplegen, Inc., Pleasanton CA, USA).

### 2.2. Screening for Bacteriocins Effective against VRE Strains

Bacteriocinogenic LAB from the culture collection of the ProBacLab (Handong Global University, Pohang, Republic of Korea) were evaluated for bacteriocinogenic activity against a selected panel of VRE strains, according to the recommendations of dos Santos et al. [21]. Bacteriocinogenic strains were cultured in 10 mL MRS broth at 37 °C for 24 h, and cell-free supernatants were obtained by centrifugation (4000× *g*, 10 min, 4 °C), heat treated at 80 °C for 10 min with the aim to deactivate potentially produced extracellular proteases and hydrogen peroxide, and the pH was adjusted to 6.0–7.0 with 1 M NaOH. Aliquots of 10 μL from the previously described cell-free supernatant were spotted on the surface of previously prepared plates containing BHI (Difco) supplemented with 1% agar and the studied VRE strains on an individual basis at a final concentration around 10^5^ CFU mL^−1^. Plates were incubated for 24 h at 37 °C and observed for the formation of inhibition zones around spotted material. Inhibition zones larger than 3 mm were considered as evidence for bacteriocinogenic activity against the VRE strains. In addition, the titer of the bacteriocin activity, expressed in AU mL^−1^, was determined according to dos Santos et al. [21]. The cell-free supernatants prepared, as described before, were serially diluted in 100 mM potassium phosphate buffer at pH 6.5, and the dilutions were spotted on the surface of the previously prepared plates with the incorporated VRE strains, as described before. The titer of bacteriocin activity expressed in AU/mL was calculated as (D^n^)/p, where D is the dilution factor used in the serial dilution, *n* is the first dilution with no inhibition zone, and *p* is the volume of the cell-free supernatant spotted in mL.

### 2.3. Partial Purification of the Bacteriocins

The selected bacteriocinogenic strains were grown in 500 mL MRS broth at 37 °C for 24 h, and the cell free supernatants were obtained and heat treated, and the bacteriocin activity (AU mL^−1^) was determined, as described before. Partial bacteriocin purification was performed, according to Todorov et al. [22], including ammonium sulfate precipitation (60% saturation) at 4 °C for 4 h with continuous shaking followed by centrifugation at 10,000× *g*, 60 min, 4 °C. The pellet was resuspended in 1/10 from the original volume in 25 mM potassium phosphate buffer pH 6.5, and the bacteriocin activity (AU mL^−1^) of the obtained suspension and supernatant was determined, as described before. In the next step, hydrophobic chromatography was performed on SepPakC18 with a step gradient of 20, 40, 60, and 80% isopropanol in 25 mM potassium phosphate buffer at pH 6.5. The obtained fractions were collected and evaluated for bacteriocin activity (AU mL^−1^), as described before, and preserved at −20 °C for further studies. All bacteriocin tests were performed against *Listeria monocytogenes* ATCC15313 and *Enterococcus faecium* VRE 9, 18, 19, and 23.

### 2.4. Determination of MIC for Vancomycin, Other Antibiotics, and Semi-Purified Bacteriocins for Enterococcus faecium Strains VRE 9, 18, 19, and 23

The antimicrobial susceptibility testing of the selected VRE strains (Cheil Jedang Pharma Co., Seoul, Republic of Korea), against vancomycin, ampicillin, chloramphenicol, gentamicin, and kanamycin (Sigma-Aldrich) was performed according to the recommendations of the Clinical and Laboratory Standards Institute (CLSI, 2016, www.clsi.org (accessed on 3 June 2021)) on Performance Standards for Antimicrobial Susceptibility Testing for *Enterococcus* spp. on cation-adjusted Mueller–Hinton broth (Sigma-Aldrich) supplemented with MRS (5.0 g L^−1^). The experimental assay was performed in a 96-well microplate (SPL Life sciences, Pocheon-si, Gyeonggi-do, Republic of Korea) and performed in 10 two-fold dilutions of the antibiotics and selected semi-purified bacteriocins (40, 60, and 80 isopropanol fractions) and controls (growth and sterility controls). The bacterial inoculums were adjusted to 0.5 McFarland units (corresponding to approximately 10^7^ CFU mL^−1^) and distributed accordingly to obtain a final concentration of 10^5^ CFU mL^−1^. The plates were incubated at 35 ± 1 °C for 18 h. Interpretation of the results was focused on the lowest concentration with complete bacterial growth inhibition and was recorded as the MIC and analyzed according to the standards set for *Enterococcus* spp. [23,24].

### 2.5. Evaluation of Proportions between Resistant and Susceptible Mutants in the Population of the VRE Strains

For the identification of resistant and susceptible *E. faecium* strain, VRE19, previously isolated from a hospital in South Korea, 100 subcultures were evaluated. The subcultures of this strain were grown on 1.5% MRS agar plates, and two mirror plates were made from the original plate, supplemented with 64 and 32 µg mL^−1^ of vancomycin, respectively. The plates were cultured for 48 h at 37 °C under aerobic conditions and based on the observed bacterial growth, the subcultures were classified as resistant if the bacteria grew just in the mirror plates supplemented with vancomycin and susceptible if the bacteria only grew on the MRS plates without added antibiotic.

### 2.6. Effect of Individual and Combined Application of Bacteriocin and Antibiotics on VRE Strains

Antimicrobials commonly used in bacterial infections therapy were tested, according to the EFSA protocol [25]. Ampicillin, chloramphenicol, gentamicin, kanamycin, and vancomycin were used in double-fold dilutions starting from 128 to 0.25 µg mL^−1^ and added to 10 mL of LSM broth (IsoSensitest broth (90%) and MRS broth (10%), adjusted to pH 6.7). The VRE strains were inoculated to the previously prepared media supplemented with antibiotics and cultured at 37 °C for 24 h under aerobic conditions. The lowest concentration of each antibiotic that inhibits the visible growth of bacteria was determined after 24 h. The susceptibility cut-off values were defined according to the EFSA standards [25].

The effect of combinations of the bacteriocin and antibiotics was determined in a 96-well microplate (SPL Life sciences), where the horizontal wells contained sequentially reduced concentrations of the tested bacteriocin (from 1600 AU mL^−1^ to 6.25 AU mL^−1^), and the vertical wells contained different concentrations of vancomycin (from 1 to 64 μg mL^−1^). The wells were supplemented with MRS seeded with the *E. faecium* VRE 9, 18, 19, and 23 strains, respectively. Over regular intervals (6, 9, 18, 21, and 24 h) bacterial growth was evaluated by changes in the OD at 600 nm determined in a SPECTROstar (BMG LABTECH, Ortenberg, Germany).

### 2.7. Fluorescent In Situ Hybridization

The hybridization method was based on the procedure of Azevedo et al. [26] and Oliviera et al. [27] with slight modifications. For all strains, the growth medium was removed by centrifuging the culture at 10,000× *g* for 5 min and resuspending in 1.5 mL of 1× PBS. The washed bacteria were collected by centrifugation at 10,000× *g* for 5 min and resuspended in 1.5 mL of 4% paraformaldehyde (pH 7.2), and incubated for 1 h at room temperature, followed by centrifugation (10,000× *g* for 5 min) and resuspension of the bacteria in 1.5 mL of 50% ethanol and subsequently incubated for 1 h at −20 °C. A total of 20 μL of the fixed bacteria at an OD_600 nm_ 0.5–0.8 was washed in 480 μL of 1× PBS and centrifuged at 10,000× *g* for 5 min. The pellet was then resuspended in the hybridization buffer of [10% (*w*/*v*)] dextran sulphate, 10 mM sodium chloride, 30% (*v*/*v*) formamide, 0.1% (*w*/*v*) sodium pyrophosphate, 0.2% (*w*/*v*) polyvinylpyrrolidone, 0.2% ficoll (*w*/*v*), 5 mM disodium EDTA, 0.1% (*v*/*v*), and 0.1% Triton X-100, 50 mM Tris-HCl) with 200 nM of the specific probe, and the bacteria were incubated at 75 °C, for 2 h. The hybridized bacteria were collected by centrifugation at 10,000× *g* for 5 min, and the pellet was resuspended in 500 μL of wash buffer pH 9.0 (5 mM Tris base, 15 mM sodium chloride, 1% (*v*/*v*) Triton-X-100) and incubated for 1 h at room temperature. After incubation, the bacteria were collected by centrifugation at 10,000× *g* for 5 min and resuspended in 200 μL DW.

### 2.8. FLOW Cytometry Analysis

A ZE5 flow cytometer (Bio-Rad, Hercules, CA, USA) was used to measure the forward scattering and fluorescence of microbial cells. These parameters were acquired as pulse height signals for 10,000 events at a rate of 1 μL per second and at a fluorescence wavelength of 647 nm. Prior to analysis, quality control was performed using ZE-series QC beads (Bio-Rad, #12004403). The instrument tubing was cleaned by sequentially using 1% bleach, 70% ethanol, and sterile PBS. Data were acquired using the Everest software package v1.4.

### 2.9. Virulence Genes in VRE

The DNA extracted, as described previously, was analyzed by PCR for the presence of the selected virulence genes (*asa1*, *cyt*, *efaA*, *esp*, *hyl,* and *IS16*), according to the recommendations of EFSA [26]. The PCR reactions were performed in a Veriti 96-well Thermal Cycler, Applied Biosystems (Thermo Scientific, Waltham, MA, USA). The primer sequences and amplification conditions were applied, according to Fugaban et al. [15]. The amplicons were separated on 1.5% (*w*/*v*) agarose gels in 1× TAE and visualized, as described before.

### 2.10. Determining the Changes in the MIC after Exposure to Low Concentrations of Bacteriocin

To evaluate the long-term effect of bacteriocin treatment on the virulence potential of VRE, the *E. faecium* strains VRE 9, 18, 19, and 23 were cultured in 10 mL MRS broth, supplemented with sublethal doses of the semi-purified bacteriocins (50 AU mL^−1^) at 37 °C for 24 h and sub-cultured consequently for 30 days. At the beginning of the experiment (day zero) and after 30 days, the MIC of the *E. faecium* VRE 9, 18, 19, and 23 strains receiving different bacteriocins were determined, according to the method described previously in this manuscript. The MICs at day zero and after 30 days were compared. The experiment was repeated on two independent occasions.

## 3. Results and Discussion

### 3.1. Differentiation and Identification of VRE Strains and Detection of the Type of Vancomycin Resistance

**Figure 1:**
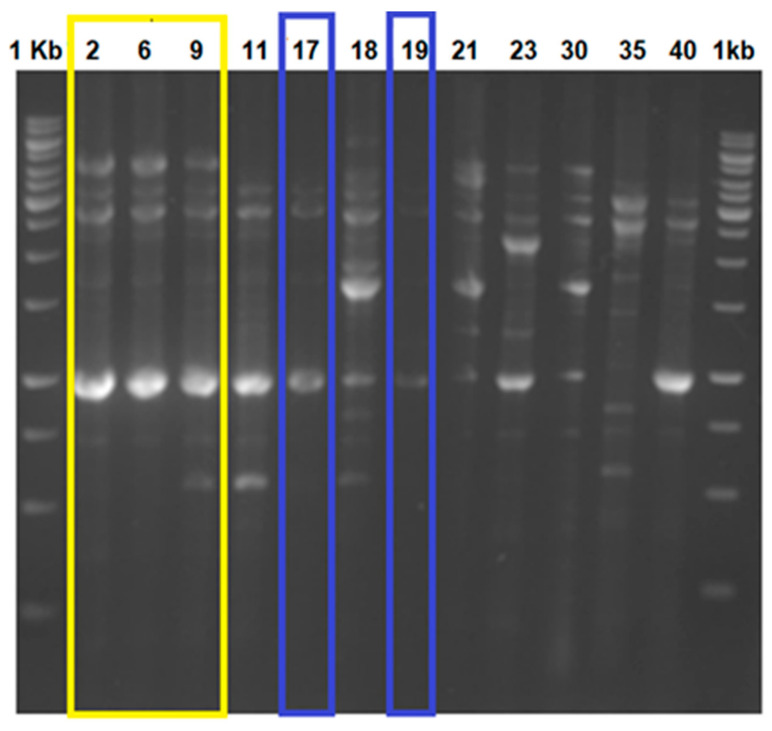
Fingerprints of the collected VRE strains from clinical origin. RepPCR was used to differentiate the strains and discard those belonging to the same group (colored boxes). The 1 kb ladder is from Fermentas.

Twelve (12) vancomycin-resistant Enterococcus (VRE) strains, previously isolated from patients, were kindly provided by Prof. Kwak (Laboratory of Antibiotics, Handong Global University, Pohang, Republic of Korea) and were differentiated by fingerprinting using repPCR (Figure 1). The unique strains were selected and identified as *E. faecium* 9, 18, 19, and 23 based on the 16S rRNA sequences (commercial service from SolGent South Korea) and by applying additional morphological, physiological, and biochemical tests recommended by Bergey’s Manual [20]. It was interesting to observe that some of the evaluated VRE isolates generated similar repPCR profiles (Figure 1), which may be evidence that the same strain has been isolated on multiple occasions This suggests that specific resistant VRE strains are well established in healthcare units. Additional studies should be performed with more clinical samples and a relevant number of isolates to confirm our preliminary hypothesis and to investigate if the mentioned strains are associated with specific healthcare units or possibly have also spread to larger regions. Interestingly, all evaluated and sequenced cultures were identified as *E. faecium*, except for VRE2, which was identified as *Enterococcus lactis*. Identification of the enterococci by 16S rRNA is not definitive, and additional biochemical and physiological tests are required. In general, vancomycin resistance is associated with *E. faecalis* [28]; however, other representatives of the genus *Enterococcus* can also be carriers of vancomycin-resistant genes.

The VRE strains selected for this study were evaluated for the presence of different vancomycin-resistance-associated genes (*van*A, *van*B, *van*C, *van*D, *van*E, and *van*G), and the predominant presence of *van*A and *van*B were recorded. Moreover, *van*D was also recorded in strain *E. faecium* VRE17. According to previous reports, vancomycin-resistant genes can be equally distributed on the bacterial chromosome and the plasmid DNA [29]. Different suggestions were hypothesized related to the relevance of the genetic locations of vancomycin-resistant genes, from serious to relatively low risk for possible gene transfer and spread around other bacterial species via horizontal and vertical gene transfer [30,31]. Moreover, Suvorov [32] discussed the relevant scenarios, such as genetic transfer in the GIT of humans and animals or in food production environments. Healthcare units, however, need to be considered as a principal environment for the relevance of VRE strains based on the extensive application of antibiotics and their role in the selection and domestication of VRE mutants.

### 3.2. Virulence Genes in Enterococcus faecium Strains VRE 8, 18, 19, and 23

A major safety concern of VRE strains has been associated with the genetic determinants for vancomycin resistance. However, several other virulence factors can be recorded in both vancomycin-resistant and non-vancomycin-resistant enterococci. The DNA extracted, as described previously, was analyzed by PCR for the presence of the selected virulence genes (cyt, *asa1*, *efa*A, *esp*, *hyl*, and *IS16*). The PCR results indicated the presence of *efa*A, *esp*, and *hyl* genes in all the tested VRE strains; yet, *cyt* and *asa1* were absent in these four strains. The virulence factor, *efa*A, is of clinical importance and has been typically associated with cases of endocarditis [33]. The protein, *esp*, is an adhesin that allows biofilm formation and colonization of epithelial cells in the urinary tract; it has been associated with VRE outbreaks, suggesting a key role in nosocomial infections [34].

Padilla and Lobos [35] reported that of 78 *Enterococcus* strains isolated from water wells for human consumption, those with the greatest number of virulence factors showed greater resistance to antibiotics. This correlation could be due to the irresponsible use of antibiotics, resulting in the selection of strains in the environment with greater pathogenicity and highlighting the importance of the use of alternatives for the control of pathogenic bacteria.

### 3.3. Screening for Bacteriocins Effective against VRE Strains

The strategy of the selection of new antimicrobials for the combat of the VRE is based on the search of inhibitory molecules that will effectively kill the target organisms by acting via a different action mechanism and that is associated with low cytotoxicity but with a specific spectrum of effective activity. In general, the development of new antibiotics aims at a broad-spectrum mode of action but may be associated with several side effects [36]. Most of the proposed new antibiotics are effective against VRE and can be applied in clinical practices. However, their broad phylogenetic spectrum generally results in the disruption of numerous beneficial gut microbiota. This may have some serious health consequences, especially with long-term applications [37]. One of the potential alternatives in the screening for potentially effective antimicrobials against VRE could be the use of bacteriocins. Based on their narrow spectrum of activity [38] and specificity in the bactericidal mode of action [9], several reports considered them as relevant candidates for clinical trials towards application as alternatives or as synergetic partners to antibiotics in the treatment of VRE-associated infections [39].

**Figure 2:**
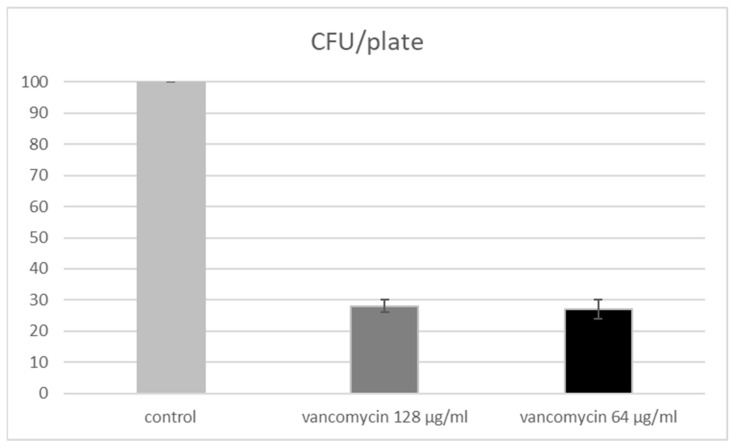
Selection of antibiotic resistance in CFU. To determine whether the isolated CFUs were VRE, mirror MRS plates were made without and with 128 µg mL^−1^ or 64 µg mL^−1^ vancomycin (final concentration). Results are the average of the four independent experiments.

In our study, we evaluated four strains, previously described as bacteriocin producers, for their effective inhibition/killing of VRE bacteria (Table 1, Figure 2). *Pediococcus acidilactici* ST3522BG, *P. pentosaceus* ST3633BG, *E. faecium* ST651ea, *E. faecium* ST7119ea, and *E. faecium* ST7319ea formerly isolated from silage and from Korean fermented food products [15,40].

The bacteriocinogenic potential of the evaluated LAB strains versus selected VRE is presented in Table 1. The comparison of the inhibitory potential versus VRE, as well taking into consideration additional bacteriocinogenic properties of the tested bacteriocins [15,40], identified three bacteriocins (produced by *P. acidilactici* ST3522BG, *P. pentosaceus* ST3633BG, and *E. faecium* ST7319ea) that were selected for future studies.

### 3.4. Partial Purification of the Bacteriocins

By a combination of precipitation with ammonium sulfate at 60% saturation followed by hydrophobic chromatography on SepPak C18 with elution with a stepwise gradient of isopropanol in 25 mM potassium phosphate buffer at pH 6.5, fractions of bacteriocins from *P. acidilactici* ST3522BG, *P. pentosaceus* ST3633BG, and *E. faecium* ST7319ea were obtained predominantly in the 60% and 80% isopropanol fractions. The combination of ammonium sulfate precipitation and hydrophobic chromatography on SpePakC_18_ can be considered a highly successful approach for the partial purification of bacteriocins produced by LAB and was applied formerly in purification protocols by Todorov et al. [22], Metivier et al. [41], Bughaloo-Vial et al. [42], Song et al. [43], and Surovtsev et al. [44]. By nature, all bacteriocins are proteinaceous, and ammonium sulfate precipitation combined with the level of saturation gives excellent results for the preliminary extraction of these proteinaceous molecules with a specific range of molecular sizes [45]. The use of hydrophobic SepPakC_18_ columns exploits the hydrophobic character of most bacteriocins. By further application of an isopropanol gradient, these antimicrobial peptides can be eluted at a specific point [44]. These approaches were previously applied in the partial purification of several bacteriocins [22,41,42,43,44]. The obtained active fractions can be applied for the purpose of analytical experiments or can be subjected to further purification, depending on the objective of the research.

### 3.5. Determination of MIC for Vancomycin and Semi-Purified Bacteriocins against Selected VRE Strains

For the determination of the MICs of the VRE strains to vancomycin and the previously prepared semi-purified bacteriocins the recommendations of CLSI were followed; the obtained results are summarized in Table 2. The purpose of this experiment was to determine the break point for the possible inhibitory application of vancomycin and semi-purified bacteriocins and to plan the experiment for the combined antibiotic–bacteriocin application. As expected, lethal concentrations of vancomycin exceeded levels of 128 μg mL^−1^ for all tested VRE strains. By contrast, even at 200 AU mL^−1^, the studied bacteriocins inhibited/killed the studied VRE strains. Similar results have formerly been reported for the lethal activity of different bacteriocins against VRE strains [19,46,47]. It is known that vancomycin uses lipid II as a docking molecule for the initial contact with target cells; however, additional steps in the antibiotic mode of action were required to effectively kill the target cells [48]. In a similar way, bacteriocins from class IIa, belonging to the two studied pediocins and the one enterocin, are associated with the recognition of lipid II and pore formation as part of the killing mode of action [49]. Comparison and similarity of the initial steps of the use of the same receptor and better effectiveness of the bacteriocins in the successful killing of VRE can be considered as a solid argument towards deepening the exploration for the potential application of bacteriocins as an alternative to conventional antibiotics in the therapy of antibiotic-resistant pathogens.

### 3.6. Effect of Bactriocins, Dead, Resistant, and Susceptible Strains in the Population of the Studied VRE Strains

The presence of antibiotics can be considered as a preselection factor for survival and predomination of mutant-carrying, antibiotic-resistant genes [50]. This is one of the reasons that the hospital environment is considered as an “incubator” for antibiotic-resistant mutants [51]. The inappropriate application of antibiotics in combination with the natural process of development of resistance into the pathogenic bacteria, the driving force of selectively killing susceptible pathogen variants, and the survival of resistant mutants, as well as the reduction of natural intraspecies competition, can lead to the establishment of predominating resistant pathogen populations [52].

**Figure 3:**
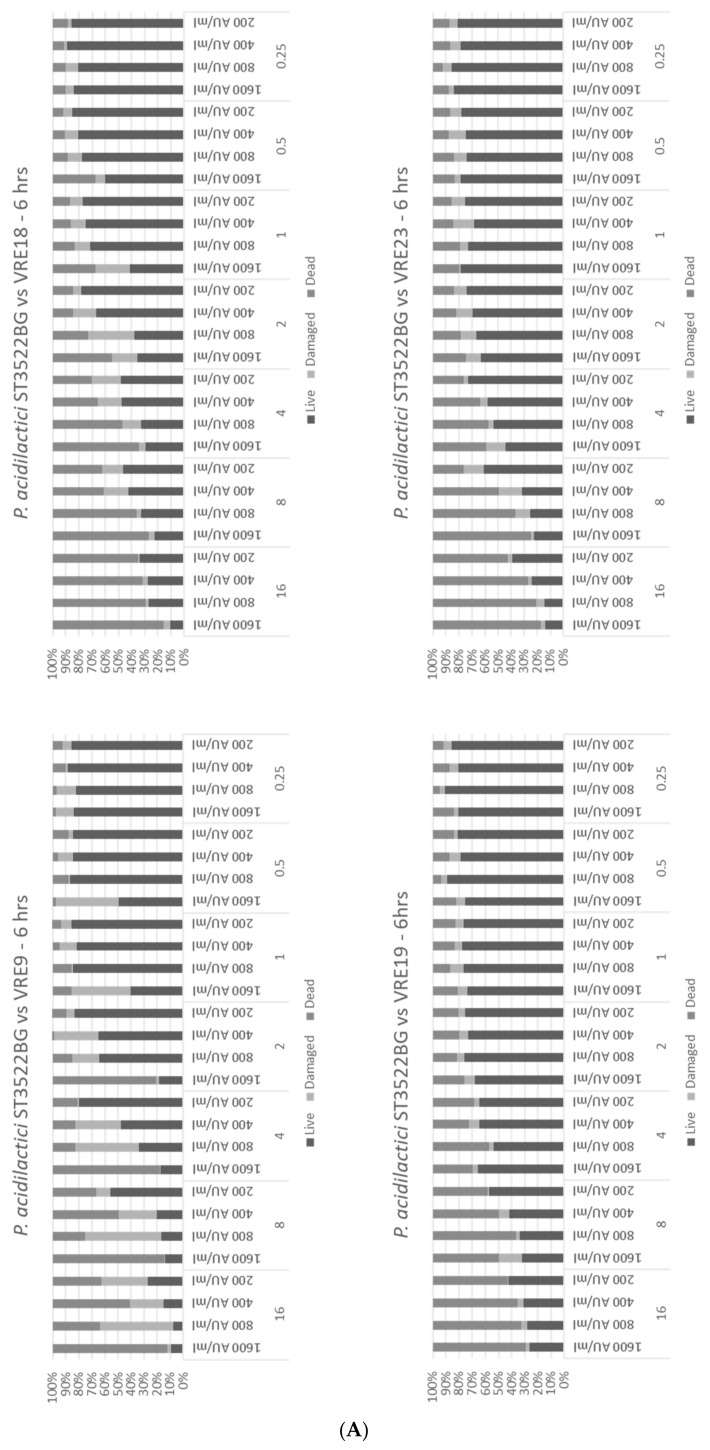

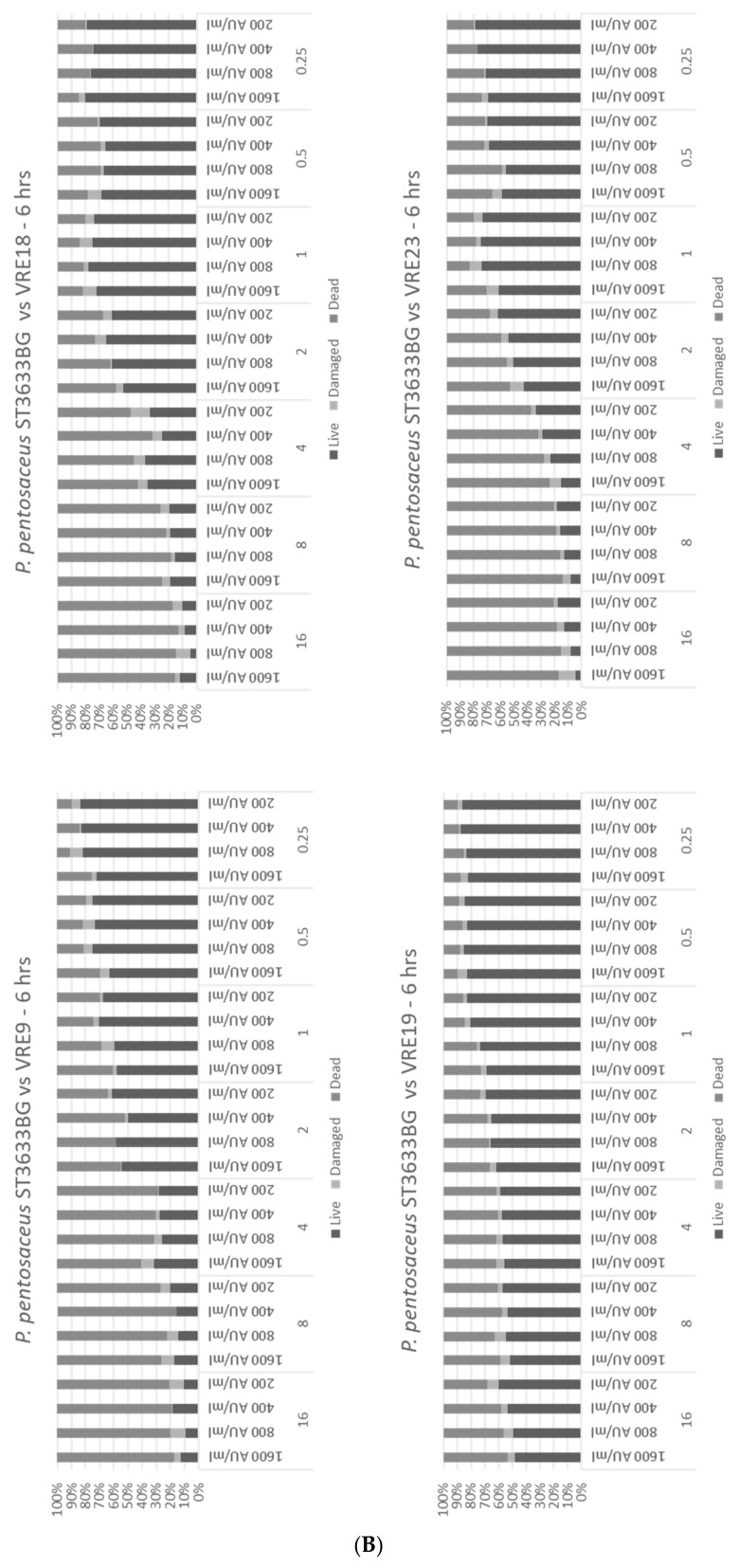

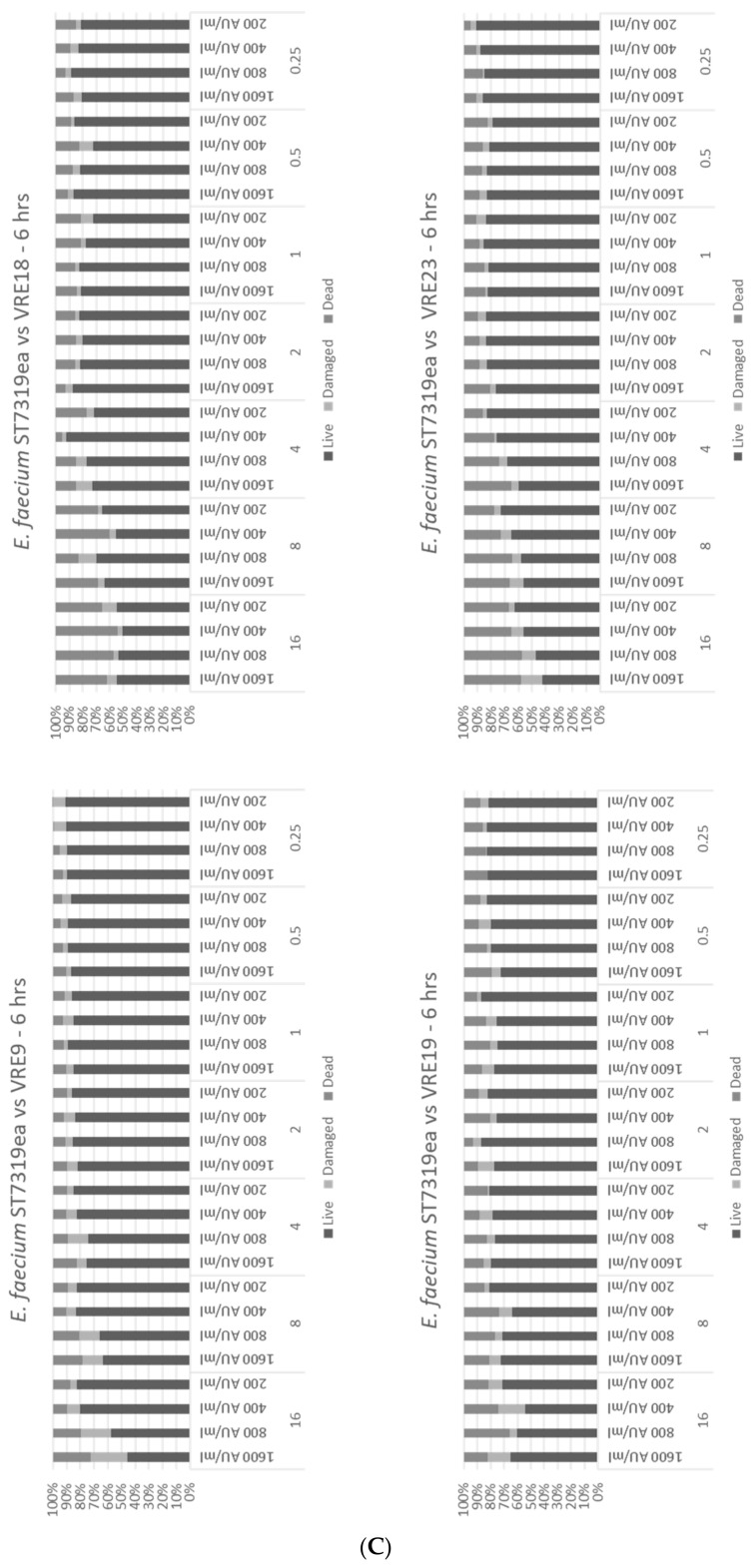
Viability test of VRE strains by flow cytometry. VRE strains (*E. faecium* VRE9, *E. faecium* VRE18, *E. faecium* VRE19, and *E. faecium* VRE23, as indicated)—were challenged with different concentrations of vancomycin between 0.5 and 16 µg mL^−1^ and bacteriocins produced by (**A**): *P. acidilactici* ST3522BG, (**B**): *P. pentosaceus* ST3633BG, and (**C**): *E. faecium* ST7319ea with concentrations between 1600 and 200 AU mL^−1^, as indicated. After 6 h incubation at 37 °C, cultures were analyzed by flow cytometry.

The analysis of the population of VRE strains showed that not all colonies could be characterized as antibiotic resistant (Figure 3). The presence of damaged cells were observed when low levels of antimicrobials were applied. Flow cytometry is an appropriate approach to evaluate dead, damaged, and live cells, and based on the last, conclusions about efficacies of applied antimicrobials can be drawn. This result was not surprising since, from a selective point of view, the absence of a lethal factor can be the driving argument for loss of the appropriate defense mechanism(s). In other words, the absence of antibiotics may result in a reduction of the defense system and decreasing levels of MIC. However, the entire process is random and does not cover all cells in the population. Always, a small part may still retain the defense mechanisms, and in return for the killing factor (antibiotic), this can be effective only against susceptible mutants, while the resistant mutants will survive and soon become predominant under a prevailing selective factor.

As previously indicated, we hypothesize that by the combined treatment with antibiotics and bacteriocins, it should be possible to kill VRE pathogens more effectively by increasing the susceptibility of the antibiotic-resistant mutants. We observed that combining the bacteriocins from *P. acidilactici* ST3522BG, *P. pentosaceus* ST3633BG, and *E. faecium* ST7319ea with vancomycin had an enhanced antimicrobial effect on the VRE strains (Figure 3) such as was the case for the bacteriocins from *P. acidilactici* ST3522BG. At the highest concentrations of bacteriocin and vancomycin, an inhibition of up to 90% was determined; when the bacteriocins were diluted to a concentration of 200 AU mL^−1^ with the same concentration of antibiotic, 60% of VRE cells were either damaged or killed (Figure 3A).

The treatment of vancomycin mixed with the semi-purified bacteriocin obtained from *E. faecium* ST7319ea resulted in a low-inhibition level of the VRE strains (Figure 3C); this could be due to both the bacteriocin and vancomycin having a similar site of action. The target of vancomycin is lipid II; coupling of vancomycin would, therefore, inhibit the synthesis of the peptidoglycan. In a similar fashion, lipid II is a docking site for nisin [53]. For this reason, it is essential to first determine the mechanism of action of this bacteriocin.

### 3.7. Effect of Individual and Combined Application of Bacteriocin and Vancomycin on VRE

The application of a combination of antibiotics and bacteriocins for the control of human and animal pathogens has been explored before. The combined application of ciprofloxacin and bacteriocin produced by *P. pentosaceus* ST44AM showed a synergistic effect against *Listeria ivanovii* subsp. *ivanovii* ATCC 19119 [54]. Moreover, the combination between ciprofloxacin and five bacteriocins produced by *Lb. plantarum* ST69BZ, *E. faecium* ST62BZ, and *L. lactis* strains, ST63BZ, ST611BZ, and ST612BZ (all isolated from boza) showed synergistic effects in combination [55]. This may be supported by the fact that pore formation by these bacteriocins could facilitate the antibiotic killing action against sensitive bacterial cells. Alternatively, the weakness of the pathogen population as a result of the combined application of two different antimicrobials with a different mode of action may result in more effective lethal activity against pathogens.

**Figure 4:**
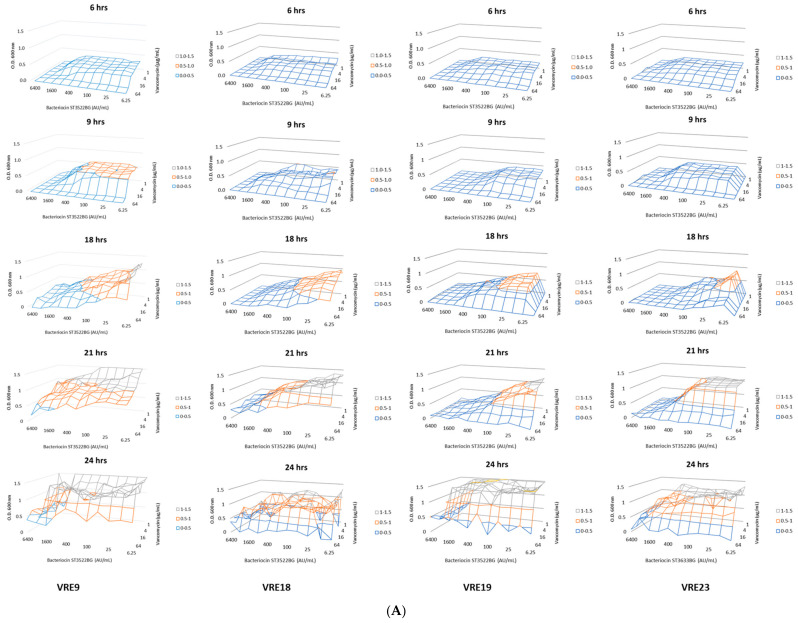

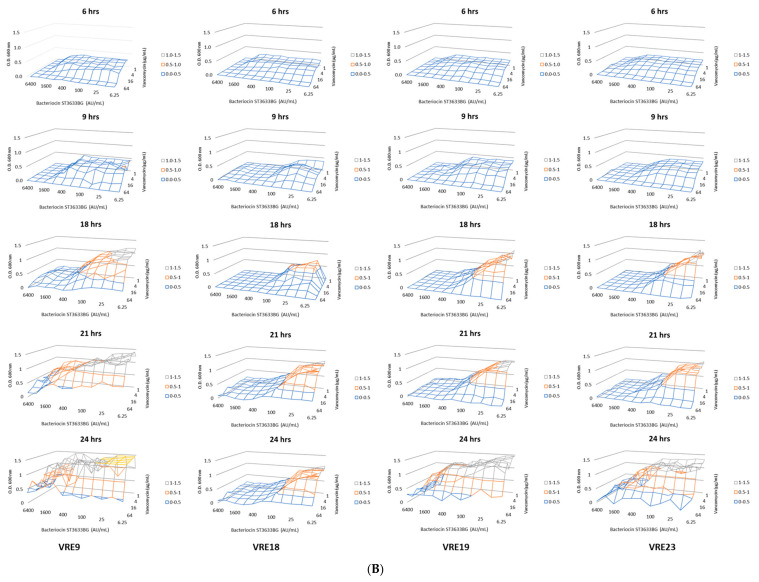

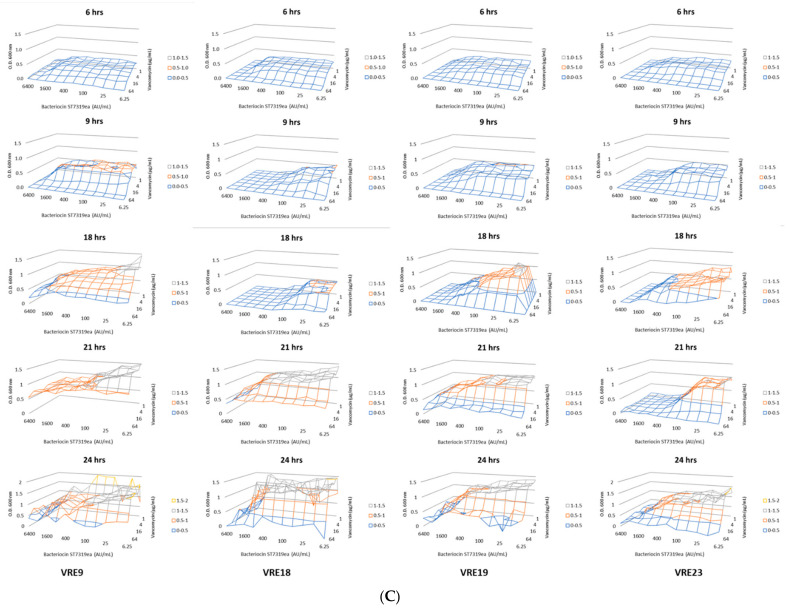
Viability test of VRE strains (*E. faecium* VRE9, *E. faecium* VRE18, *E. faecium* VRE19, and *E. faecium* VRE23, as indicated) by spectrophotometry. VRE strains were challenged with different concentrations of vancomycin (1.0 to 64 µg mL^−1^) and bacteriocins produced by (**A**): *P. acidilactici* ST3522BG, (**B**): *P. pentosaceus* ST3633BG, and (**C**): *E. faecium* ST7319ea (6.25 to 6400 AU mL^−1^). Cultures were incubated for 24 h and analyzed at 6, 9, 18, 21, and 24 h by spectrophotometry, as indicated on the figures.

The combined application of vancomycin and semi-purified bacteriocins obtained from *P. acidilactici* ST3522BG, *P. pentosaceus* ST3633BG, and *E. faecium* ST7319ea showed a possible synergistic interaction and improved the inhibition of the VRE test strains. Moreover, the killing effect was observed by following the bacterial growth of VRE exposed to different combinations of vancomycin (from 0.25 to 128 μg mL^−1^) and bacteriocins produced by *P. acidilactici* ST3522BG, *P. pentosaceus* ST3633BG, and *E. faecium* ST7319ea (from 200 to 1600 AU mL^−1^) (Figure 4A–C). The destruction effect by applying the Fish-Flow approach, was the detection of live, damaged, and dead cells; these results complemented previous observations by monitoring the bacterial growth (Figure 3A–C).

The evaluation of the combined effect of antibiotics and bacteriocins by Fish-Flow is a novel approach; it complements the previously explored microtiter plates for evaluation of bacterial growth, such as VRE or other relevant pathogens, when exposed to a combination of antibiotics and bacteriocins or other antimicrobials. The Fish-Flow approach can be considered as more precise since it clearly distinguishes between the dead, damaged, or living cells after treatment, respectively. Thereby, more clarity is provided on the effect of combined applications of antimicrobials, resulting in bactericidal, bacteriostatic, or no effect on the test organisms. Moreover, the use of specific probes can increase the reliability of the results because the obtained results will clearly represent the viability of the targeted test organisms for which the probes were designed. Thereby, misinterpretations of the results can be reduced, e.g., in case of potential bacterial contamination. Moreover, a possible clinical application may be to evaluate the effect of different ratios of antibiotics and bacteriocins (or other antimicrobials or even including a combination of antibiotics with a different mode of action) on fecal samples from patients diagnosed with VRE infections. This may enable the prediction of appropriate proportions of different antimicrobials for the effective treatment against the elimination of pathogens.

### 3.8. Following the Changes in the MICs after Exposure to Low Concentrations of Bacteriocin

It is possible that the continuous exposure of bacteria to bacteriocins could result in the selection of innate or acquired resistance of bacteria, as has already been reported for conventional antibiotics [56]. To study the possible acquisition of resistance of the VRE strains against bacteriocins, a long-term application was carried out where no significant change in the MICs was observed during the experimental period (Table 2). It was observed that the VRE inhibition levels after 30 days of exposure to bacteriocins were the same or very similar to those obtained on day 0, as was also observed in previous experiments.

This could indicate that the VRE strains studied do not have innate mechanisms of resistance to these bacteriocins. However, it is necessary to study the models for a longer period of time to know in detail the possible mutations that may cause the acquisition of a resistance mechanism against these molecules.

## 4. Conclusions

The necessity for new effective antimicrobials against emerging antibiotic-resistant pathogens is a fact and has become an issue of increasing importance since the beginning of this century. Bacteriocins, as part of the group of antimicrobial peptides, can be considered as an alternative and/or complement to known antibiotics. Our study reaffirms the importance of bacteriocins in the control of pathogenic bacteria. Moreover, our results suggest a possible synergistic effect of bacteriocins when combined with vancomycin, which offers a new approach of combat antibiotic resistance; the specific spectrum of activity would make them a good option for the treatment of already diagnosed specific infections and in the case of the food industry, thus, helping to inhibit the proliferation of resistant bacteria. Although more studies are needed to characterize these bacteriocins and their spectrum of activity in detail, we consider them to be a reliable option for the control of pathogenic microorganisms.

## Figures and Tables

**Table 1:** Evaluation of bacteriocinogenic potential against clinical *Enterococcus faecium* VRE strains. The selected bacteriocin produced by *P. acidilactici* ST3522BG, *P. pentosaceus* ST3633BG, *E. faecium* ST7119ea, *E. faecium* ST651ea, and *E. faecium* ST7319ea were tested versus the investigated VRE strains. Growth inhibition was presented by measuring the inhibition zones, expressed in millimeters. (-): no growth inhibition. *L. monocytogenes* ATCC 15313 and *L. innocua* ATCC 33090 served as controls. Results are average values from three independent repetitions.

Species	Strain ID	Bacteriocin (mm Inhibition Zones)				
		*P acidilactici* ST3522BG	*P. pentosaceus* ST3633BG	*E. faecium* ST7119ea	*E. faecium* ST651ea	*E. faecium* ST7319ea
*E. lactis*	VRE2	-	-	-	-	-
*E. faecium*	VRE6	14	18	-	-	6
	VRE9	15	16	-	-	-
	VRE17	15	16	15	9	9
	VRE21	14	14	-	-	-
	VRE35	12	14	-	-	6
	VRE40	13	15	11	13	15
	VRE18	14	17	12	14	7
	VRE23	17	19	14	14	15
	VRE11	16	18	16	17	16
	VRE19	15	17	12	14	15
	VRE30	14	15	13	14	14
*L. monocytogenes*	ATCC15313	14	17	-	4	5
*L. innocua*	ATCC33090	17	18	15	15	17

**Table 2:** Long-term exposition of selected VRE strains to the bacteriocins produced by *P. acidilactici* ST3522BG, *P. pentosaceus* ST3633BG, and *E. faecium* ST7319ea. The test organisms were grown in the MRS supplemented with 50 AU mL^−1^ (final concentration) for 30 days and at 37 °C, and consequently, MIC was evaluated. In bold were highlighted VRE strains and antibiotic’s MIC from initial screening applied in long term exposure to sublethal doses of studied bacteriocins.

VRE Strain	Ampicillin	Chloramphenicol	Gentamicin	Kanamycin	Vancomycin
Initial MIC (before Exposure to Sublethal Doses of Bacteriocins)					
2	128	16	≥128	≥128	≥128
6	64	2	≥128	128	≥128
**9**	**128**	**4**	**≥128**	**≥128**	**≥128**
11	64	4	≥128	≥128	≥128
17	64	4	≥128	≥128	≥128
**18**	**32**	**8**	**≥128**	**≥128**	**≥128**
**19**	**32**	**4**	**≥128**	**≥128**	**≥128**
21	32	4	≥128	≥128	≥128
**23**	**128**	**32**	**≥128**	**≥128**	**≥128**
30	32	4	16	64	128
35	64	8	≥128	≥128	≥128
40	64	8	32	64	64
MIC after Exposure to Bacteriocin Produced by ***P. acidilactici* ST3522BG**					
9	**128**	**4**	**≥128**	**≥128**	**≥128**
18	**32**	**8**	**≥128**	**128**	**≥128**
19	**32**	**4**	**≥128**	**≥128**	**≥128**
23	**128**	**16**	**≥128**	**128**	**≥128**
MIC after exposure to bacteriocin produced by ***P. pentosaceus* ST3633BG**					
9	**128**	**4**	**≥128**	**128**	**≥128**
18	**32**	**8**	**≥128**	**≥128**	**≥128**
19	**32**	**4**	**≥128**	**≥128**	**≥128**
23	**128**	**16**	**≥128**	**128**	**≥128**
MIC after exposure to bacteriocin produced by ***E. faecium* ST7319ea**					
9	**128**	**4**	**≥128**	**≥128**	**≥128**
18	**32**	**8**	**≥128**	**≥128**	**≥128**
19	**32**	**4**	**≥128**	**128**	**≥128**
23	**128**	**8**	**≥128**	**128**	**≥128**

## Data Availability

All data associated with this paper are available upon request.
